# Genomic Epidemiology of *Salmonella enterica* Serotype Enteritidis based on Population Structure of Prevalent Lineages

**DOI:** 10.3201/eid2009.131095

**Published:** 2014-09

**Authors:** Xiangyu Deng, Prerak T. Desai, Henk C. den Bakker, Matthew Mikoleit, Beth Tolar, Eija Trees, Rene S. Hendriksen, Jonathan G. Frye, Steffen Porwollik, Bart C. Weimer, Martin Wiedmann, George M. Weinstock, Patricia I. Fields, Michael McClelland

**Affiliations:** University of Georgia, Griffin, Georgia, USA (X. Deng);; University of California Irvine, Irvine, California, USA (P.T. Desai, S. Porwollik, M. McClelland);; Cornell University, Ithaca, New York, USA (H.C. den Bakker, M. Wiedmann);; Centers for Disease Control and Prevention, Atlanta, Georgia, USA (M. Mikoleit, B. Tolar, E. Trees, P.I. Fields);; Technical University of Denmark, Lyngby, Denmark (R.S. Hendriksen);; US Department of Agriculture, Athens, Georgia, USA (J.G. Frye);; University of California Davis, Davis, California, USA (B.C. Weimer);; Washington University School of Medicine, St. Louis, Missouri, USA (G.M. Weinstock);

**Keywords:** Salmonella enterica serotype Enteritidis, genome sequencing, SNP, genomic epidemiology, subtyping, evolution, bacteria

## Abstract

*Salmonella enterica* serotype Enteritidis is one of the most commonly reported causes of human salmonellosis. Its low genetic diversity, measured by fingerprinting methods, has made subtyping a challenge. We used whole-genome sequencing to characterize 125 *S*. enterica Enteritidis and 3 *S. enterica* serotype Nitra strains. Single-nucleotide polymorphisms were filtered to identify 4,887 reliable loci that distinguished all isolates from each other. Our whole-genome single-nucleotide polymorphism typing approach was robust for *S. enterica* Enteritidis subtyping with combined data for different strains from 2 different sequencing platforms. Five major genetic lineages were recognized, which revealed possible patterns of geographic and epidemiologic distribution. Analyses on the population dynamics and evolutionary history estimated that major lineages emerged during the 17th–18th centuries and diversified during the 1920s and 1950s.

## Abstract 2

Major lineages emerged during the 17th–18th centuries and diversified during the 1920s and 1950s.

*Salmonella enterica* causes ≈1 million illnesses and >350 deaths annually in the United States (*1*). Among >2,500 known serotypes, *S. enterica* serotype Enteritidis is one of the most commonly reported causes of human salmonellosis in most industrialized countries (*2*). From the 1970s through the mid-1990s, the incidence of serotype Enteritidis infection increased dramatically; shelled eggs were a major vehicle for transmission. Despite a decrease in serotype Enteritidis infection since 1996 in the United States, outbreaks resulting from contaminated eggs continue to occur (*3*), and Enteritidis remains among the most common serotypes isolated from humans worldwide (*2*). Epidemiologic surveillance and outbreak investigation of microbial pathogens require subtyping that provides sufficient resolution to discriminate closely related isolates. Differentiation of *S. enterica* Enteritidis challenges traditional subtyping methods, such as pulsed-field gel electrophoresis (PFGE), because isolates of serotype Enteritidis are more genetically homogeneous than are isolates of many other serotypes (*4*,*5*). Among the serotype Enteritidis isolates reported to PulseNet, ≈45% display a single PFGE *Xba*I pattern (JEGX01.0004), which renders PFGE ineffective in some investigations (*5*). Of the second-generation methods evaluated for *S. enterica* Enteritidis subtyping, multilocus variable number–tandem repeat analysis offers slightly better discrimination, but differentiating common patterns remains a substantial problem (*6*). Therefore, new methods are needed to better subtype and differentiate this serotype. Recent applications of whole-genome sequencing (WGS) have demonstrated exceptional resolution that enables fine delineation of infectious disease outbreaks (*7*–*10*).

In addition to sufficient subtyping resolution, accurately ascribing isolates to epidemiologically meaningful clusters, i.e., grouping isolates associated with an outbreak while discriminating unrelated strains, is critical for pathogen subtyping. Outbreak and epidemiologically unrelated isolates might not be differentiated by using current methods. Despite the high incidence of *S. enterica* Enteritidis infection in humans, genome sequencing of this serotype has lagged behind sequencing of other major foodborne pathogens. To our knowledge, only 1 finished *S. enterica* Enteritidis genome is publicly available (*11*). Recent sequencing of *S. enterica* Enteritidis genomes of the common PFGE *Xba*I pattern JEGX01.0004 has provided a valuable resource on the *S. enterica* Enteritidis genome (*12*). Here we present a broad sampling of WGS to include diversity of other major lineages.

We expanded the genomic population structure of *S. enterica* Enteritidis by sequencing a collection of 81 *S. enterica* Enteritidis genomes and 3 *S. enterica* serotype Nitra genomes selected to capture epidemiologic and phylogenetic diversity in current domestic and international serotype Enteritidis populations. We included serotype Nitra in the study because it is thought to be a variant of serotype Enteritidis with its O antigen (serogroup O2) being a minor genetic variant of serogroup O9 found in serotype Enteritidis (*13*). These genomes, along with 44 draft genomes of *S. enterica* Enteritidis (14 historical strains and 30 isolates selected from the 2010 egg outbreak investigation [http://www.cdc.gov/salmonella/enteritidis/]), provided a phylogenetic framework of diverse circulating serotype Enteritidis lineages. Model-based Bayesian estimation of age and effective population size of major *S. enterica* Enteritidis lineages showed that the spreading of *S. enterica* Enteritidis coincided with 2 periods: the 18th century period of colonial trade and the 20th century period of agricultural industrialization. A single-nucleotide polymorphism (SNP) pipeline was developed for high-throughput whole-genome SNP typing and was robust for combining data from different sequencing platforms in the same analysis. This enabled retrospective investigation of recent clinical cases in Thailand and the shelled eggs outbreak in the United States. The ability of whole-genome SNP typing to infer the polyclonal genomic nature of at least some *S. enterica* Enteritidis strains causing outbreaks, despite high genetic homogeneity among *S. enterica* Enteritidis genomes, demonstrates the utility and sensitivity of whole-genome SNP typing in epidemiologic surveillance and outbreak investigations. Potential challenges of whole-genome SNP typing, such as ways to accurately define individual outbreaks, were discussed.

## Methods

### Isolates

We obtained 125 serotype Enteritidis and 3 serotype Nitra isolates from Centers for Disease Control and Prevention, US Department of Agriculture, and University of California Davis (Technical Appendix Tables 1, 2). *S. enterica* Enteritidis isolates of diverse PFGE subtypes (18 *Xba*I patterns accounting for >90% of all *S. enterica* Enteritidis isolates reported to PulseNet [Technical Appendix Figure 1]), spatiotemporal origins, and sources were sampled to span a broad epidemiologic and phylogenetic diversity of prevalent lineages of which we were aware.

### WGS

Bacterial strains were grown in Luria broth at 37°C to stationary phase. Genomic DNA was prepared by using the GenElute Genomic DNA isolation kit (Sigma-Aldrich, St. Louis, MO, USA). Eighty-one isolates were sequenced by using Illumina (San Diego, CA, USA) technology (100-bp paired-end reads) at Washington University (St. Louis, MO, USA). Another 44 isolates were sequenced by using Roche (Indianapolis, IN, USA) 454 technology (single-end reads) as described previously (*12*).

### SNP Detection

We developed a bioinformatics pipeline to detect high-quality SNPs from raw sequencing reads. The design of the workflow was geared toward a customizable and robust solution for whole-genome SNP typing of many isolates. It enables user-defined parameters for SNP quality filters and provides additional functions, such as assembly of unmapped reads and functional annotation of SNPs (Technical Appendix Figure 2). The program snp-sites was then used to code missing data and SNP sites from ambiguous sites within the consensus sequences and create an alignment containing variable sites (https://github.com/andrewjpage/snp_sites).

### Phylogenetic Analyses

We used BratNextGen (*14*) to detect recombination events in the genomes. The consensus sequences were used as input with 100 replicates (10 iterations each) to infer the significance of detected recombination events. Regions with a significant signal of recombination were excluded, as were highly homoplastic sites (as inferred in PAUP 4.0b10 [Sinauer Associates, Inc., Sunderland, MA, USA]; rescaled consistency index <1) indicative of nonneutral evolution, recombination, or ambiguous SNP calls. The remaining SNP sites were used only for further analysis when unambiguously called for at least 95% of the isolates. We performed maximum-likelihood (ML) analyses in MEGA5.1 (*15*). The resulting ML trees were used to test for a temporal signal by using Path-O-Gen v1.3 (http://tree.bio.ed.ac.uk/software/pathogen/). Bayesian phylogenetic analyses were performed by using BEAST v. 1.7.5 (*16*). The isolation year of each isolate was used to establish a temporal framework for constructing phylogenetic relationship among the isolates and estimating parameters to describe the evolutionary dynamics of the population (*17*). Comparisons of different molecular clock models and tree priors were performed similarly to a method of Bakker et al. (*18*), except that we used the path sampling method (*19*) to estimate the marginal likelihood (Technical Appendix Table 4).

## Results

### Divergent Isolates and Serotypes

Comparison of 84 newly sequenced genomes to the reference genome showed that all but 4 were closely related to the reference strain, differing by no more than 950 SNPs. The 4 divergent genomes (77–0915, 07–0056, SARB17, and SARB19) contained 19,800–43,544 SNPs, comparable to the number of SNPs between phylogenetically distinct serotypes. They also lacked *sdf*, a characteristic marker of commonly circulating serotype Enteritidis organisms, and were phylogenetically apart from the main serotype Enteritidis lineage (Technical Appendix Figure 3). We did not include these divergent genomovars (genetic lineages) in subsequent SNP and phylogenetic analyses. The 3 *S. enterica* Nitra genomes were highly similar to the reference genome, with numbers of SNPs comparable to those of other *S. enterica* Enteritidis strains.

### High-Quality Core Genome SNPs and Phylogeny

We observed 6,542 SNP loci in the remaining strains after we excluded other genomovars. The pairwise homoplasy index test (*20*) found no evidence of recombination for the SNP data of both the 81-isolate set (Illumina sequenced isolates and the reference) and the 125-isolate set (Illumina and 454 sequenced isolates plus the reference). However, putative regions (14 in total, Technical Appendix Table 3) involved in homologous recombination were detected by BratNextGen (*14*) in the 125-isolate set, comprising 1,519 SNPs. After exclusion of these regions encompassing recombination, and homoplastic sites (136 SNPs, identified by using PAUP 4.0), 4,887 core genome SNP loci were left to be included in the analysis.

**Figure 1:**
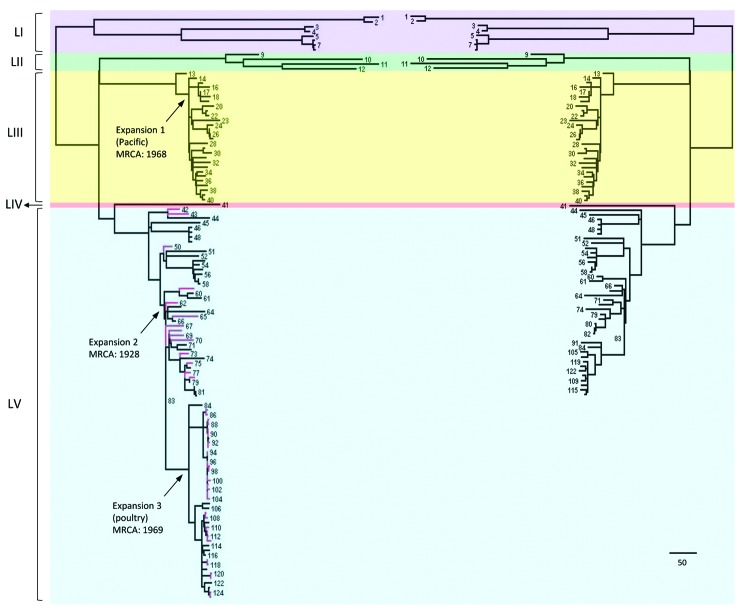
Comparison of *Salmonella enterica* serotype Enteritidis phylogenies inferred from Illumina data and combined data of Illumina (San Diego, CA, USA) and Roche 454 (Indianapolis, IN, USA). The tree on the right incudes 80 Illumina sequenced isolates and the reference genome (PT4). The tree on the left includes both the 80 Illumina and the 44 454 sequenced isolates in addition to the reference. Isolates were numbered (online Technical Appendix Table 1, http://wwwnc.cdc.gov/EID/article/20/9/13-1095-Techapp-s1.pdf). Lineages I, II, III, IV, and V are highlighted in purple, green, yellow, red, and blue, respectively. Branches representing 454 sequenced isolates are labeled in red. Arrows on the left tree indicate the 3 serotype Nitra isolates. MRCA, most recent common ancestor. Scale bar indicates 10 single-nucleotide polymorphisms.

The general time-reversible model of nucleotide substitution was the best fit model for the dataset and was subsequently used in phylogenetic analyses. ML analysis based on high-quality core genome SNPs yielded highly congruent phylogenies between the 81-isolate (Illumina data only) and the 125-isolate (Illumina and 454 data combined) datasets (Figure 1). All 454 sequenced isolates clustered in 1 lineage, including the 30 selected for the shelled eggs outbreak investigation. These isolates represented 8 of the 9 clades defined by Allard et al. (*12*). For the Illumina-sequenced isolates in both datasets, the inferred phylogenies were highly congruent (Figure 1). Five major genetic lineages were identified (Figure 1): LI, LII, LIII, LIV, and LV. Isolates from clinical cases in Thailand and associated with a shell egg outbreak in the United States were found predominately in LIII and LV, respectively.

### Population Dynamics

The 125-isolate set displayed a temporal signal, as demonstrated by a positive correlation between distances to the most recent common ancestor (MRCA) and dates of sampling. Although this correlation was weak when measured for the whole dataset (correlation coefficient 0.3, R^2^ = 0.09, p<0.001), exclusion of LII from the dataset led to an increased correlation (correlation coefficient 0.6, R^2^ = 0.40, p<0.0001). Both the Path-O-Gen analysis and molecular clock–based analyses indicate that LII evolved at a higher mutation rate than other clades.

We compared 4 population genetics models through Bayes factors (BF) (*21*). For this analysis, we excluded redundant isolates that were derived from the same outbreak and differed by only 1 or 2 SNPs. The final dataset comprised 99 isolates. A relaxed log-normal molecular clock was strongly favored over a strict clock rate (log_10_ BF>100), suggesting that mutation rates vary significantly among branches (Technical Appendix Table 4). We found strong evidence (log_10_ BF>100) in favor of a constant effective population size model over an effective population size model that enables fluctuations of effective population size through time (Gaussian Markov random field skyride model [*22*]). The results of the analyses assuming a constant effective population size model are thus discussed here.

**Figure 2:**
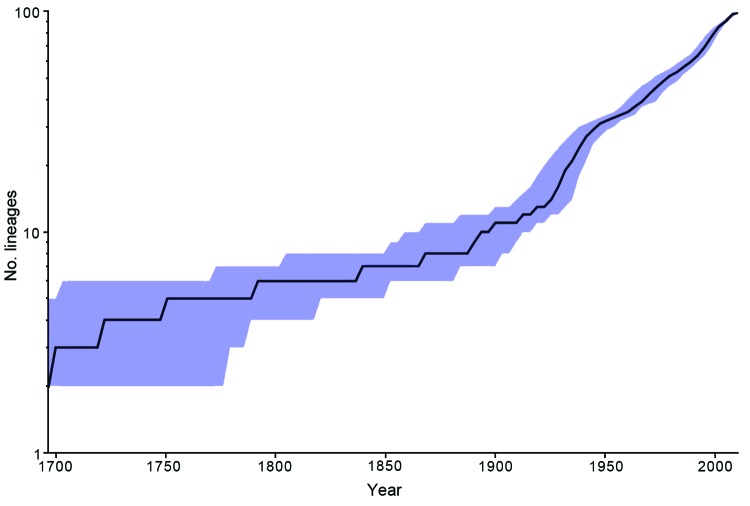
Inferred number of *Salmonella enterica* serotype Enteritidis lineages over time based on a constant effective population size model using BEAST (*16*). Blue shading indicates 95% CIs.

The mean mutation rate across all lineages was inferred to be 2.2 × 10^−7^ substitutions per site per year or 1.01 SNPs per genome per year. The MRCA of the whole population (Table) was estimated to date to 1549 ce (95% highest posterior probability density, from 1351 to 1704) (*23*). Although the inferred ages of the MRCA differ because of the model of choice, the estimates for the younger nodes appeared to converge between models, with overlapping highest posterior probability densities. We constructed a lineage through time plot (Figure 2) to show the change of inferred number of lineages over time on the basis of a constant effective population size model using BEAST (http://tree.bio.ed.ac.uk/).

**Table:** Dating of nodes in the maximum-likelihood tree of *Salmonella enterica* serotype Enteritidis using BEAST and strict and relaxed mutation rates*

Node, cluster	Median MRCA (95% CI)		
	Relaxed clock		Strict clock, constant population size
	Constant population size	GMRF	
MRCA, node N01	1549 (1351–1704)	1896 (1858–1933)	1520 (1455–1576)
MRCA II, III, IV, V	1709 (1608–1788)	NA	1680 (1636–1716)
MRCA II	1831 (1760–1897)	1937 (1903–1966)	1824 (1799–1846)
MRCA III	1941 (1919–1957)	1950 (1933–1962)	1934 (1924–1944)
MRCA V	1888 (1858–1911)	1917 (1900–1936)	1883 (1869–1897)
Expansion 1, Pacific	1968 (1958–1976)	1975 (1964–1982)	1964 (1958–1970)
Expansion 2	1928 (1916–1937)	1934 (1926–1943)	1926 (1919–1933)
Expansion 3, poultry	1969 (1956–1981)	1984 (1974–1995)	1970 (1964–1976)
*BEAST (*16*). MRCA, most recent common ancestor; GMRF, Gaussian Markov random field; NA, not available.			

## Discussion

WGS of the 125 *S. enterica* isolates of serotypes Enteritidis and Nitra enabled us to probe the population dynamics and evolutionary history of prevalent serotype Enteritidis lineages. The inferred mean mutation rates of serotype Enteritidis are comparable to those of short-term evolution in several other pathogens (*24*–*26*). The prediction that the MRCA possibly emerged between 1351 and 1704 ce is in line with the historical fact that serotype Enteritidis was one of the first recognized *Salmonella* serotypes in 1888 ce (*27*). According to the same model, the 5 lineages identified in this study originated during 1608–1788 ce (Table). This initial diversification event could possibly be related to the emergence of colonial trade around that time, facilitating effective global dispersal of serotype Enteritidis, as in the case of the intercontinental transmission of the yellow fever virus (*28*).

On the basis of the molecular clock and other assumptions made in the Bayesian analysis, the number of lineages of *S. enterica* Enteritidis is estimated to have increased sharply during 1925–1950 ce, which indicated rapid diversification of serotype Enteritidis (Figure 2). This period coincides with the Green Revolution, a period of increased global agricultural production. We speculate that the start of better poultry farming practices in the United Kingdom and United States might have created poultry farms as a niche for *S. enterica* Enteritidis by reducing closely related traditional avian *Salmonella* serotypes, such as Gallinarum (*29*). 

The genes responsible for serotype (primarily *rfb* region, *fliC*, and *fljB*) are commonly subject to horizontal gene transfer, resulting in very similar/the same alleles present in distinct genetic backgrounds. This phenomenon has contributed to the huge diversity of *Salmonella* serotypes and dictates that the serotyping system sometimes poorly reflects true phylogeny. Multiple genomovars have been noted for some serotypes (*30*) and most likely arose from horizontal gene transfer of the same assortment of serotype antigen genes into distinct genetic lineages by coincidence. Four isolates serotyped as *S. enterica* Enteritidis but lacking *sdf*, a characteristic marker of commonly circulating *S. enterica* Enteritidis, were divergent by WGS; they putatively represent different genomovars. The divergent *S. enterica* Enteritidis lineages are rarely encountered in the United States.

In contrast to the observation of different genomovars within a single serotype, *S. enterica* Nitra represents a separate serotype that is embedded within serotype Enteritidis lineages. Three serotype Nitra isolates clustered with LIII (CDC-STK-1280 and 96–0186) and LIV (2010K-0860), indicating that they are members of these *S. enterica* Enteritidis lineages. This finding is consistent with the finding that serogroup O2 is a minor genetic variant of serogroup O9. The 3* S. enterica* Nitra isolates originated from different geographic locations and decades apart, suggesting that rare, independent, and distinct mutational events caused the switch of serotype from Enteritidis to Nitra. In this instance, it seems appropriate to consider *S. enterica* Nitra as part of the *S. enterica* Enteritidis lineage. As scientists move toward genetic determination of serotype, *S. enterica* Nitra is likely to be identified as *S. enterica* Enteritidis (*13*).

As shown in this and previous studies, whole-genome SNP typing provides both superior subtyping resolution and phylogenetic accuracy without compromising either, which is rarely possible with traditional molecular subtyping methods. Whole-genome SNP typing achieves this resolution by surveying point mutations across entire genomes rather than by targeting relatively few polymorphic sites characteristic of either high mutation rates for sufficient discriminatory power (e.g., variable number tandem repeats [*31*] and clustered regularly interspaced short palindromic repeats [*32*]) or reliable phylogenetic signals for accurate phylogeny (e.g., housekeeping genes). We found that the 4,887 SNPs resulting from combining data from 2 platforms were sufficient to resolve every serotype Enteritidis isolate in a phylogeny highly congruent with the tree based only on 1 platform (Figure 1). Such robustness facilitates the use of different sequencing platforms in actual surveillance and investigations to achieve repeatable results in subtype differentiation. Specifically, the same analytical approach and bioinformatics pipeline was applied to sequencing data with drastically different error patterns by employing strict criteria to search for high-quality SNPs. This strategy was effective and efficient for phylogenetic inference, subtyping, and routine investigation of serotype Enteritidis, especially when multiple instruments, library preparation, and bioinformatics methods are available for whole-genome SNP typing applications in public health laboratories.

WGS of *S. enterica* Enteritidis isolates with a wide range of genetic, spatiotemporal, and source attributes broadened the understanding of phylogenetic diversity of this genetically homogeneous pathogen. WGS enabled us to build a phylogenetic framework of prevalent serotype Enteritidis lineages that will be highly valuable for ongoing and future surveillance and investigation.

We recognized lineages and clades with geographic and epidemiologic characteristics. Of the 3 seemingly rare or potentially undersampled lineages (LI, LII, and LIV), LI and LII appeared to be associated with specific geographic locations and have diverged earlier than other lineages. LI was further divided into 2 clades, of which one had 2 isolates from Africa (isolates 1 and 2 in Figure 1) and the other had 6 isolates from the western United States and predominately associated with wildlife and environmental sources (isolates 3–8). LII consisted of 3 isolates associated with marine mammals in California and 1 isolate linked to a human in the same state (isolates 9–12). The 3 marine mammal isolates formed a highly supported clade and came from 2 different host species in a 10-year span, suggesting a stable *S. enterica* Enteritidis lineage circulating among those animals. The fact that a human case was linked to this clade suggests possible transmission between marine animals and humans. The marine animal community on and around the Channel Islands off the coast of southern California is extremely rich and diverse and displays the highest known prevalence of *S. enterica* in the world (*33*). Free-ranging and migratory animals and birds from this natural reservoir of *S. enterica* could play a role in long distance dispersal of this pathogen. LIII and LV appeared to be the more prevalent lineages on the international and US domestic scales, respectively. LIII contained isolates from 4 continents; its major clade (isolates 14–40) originated primarily from the Pacific region, including Thailand and California. LIV represented a common lineage in the United States, including a clade predominately associated with poultry products (isolates 84–125).

**Figure 3:**
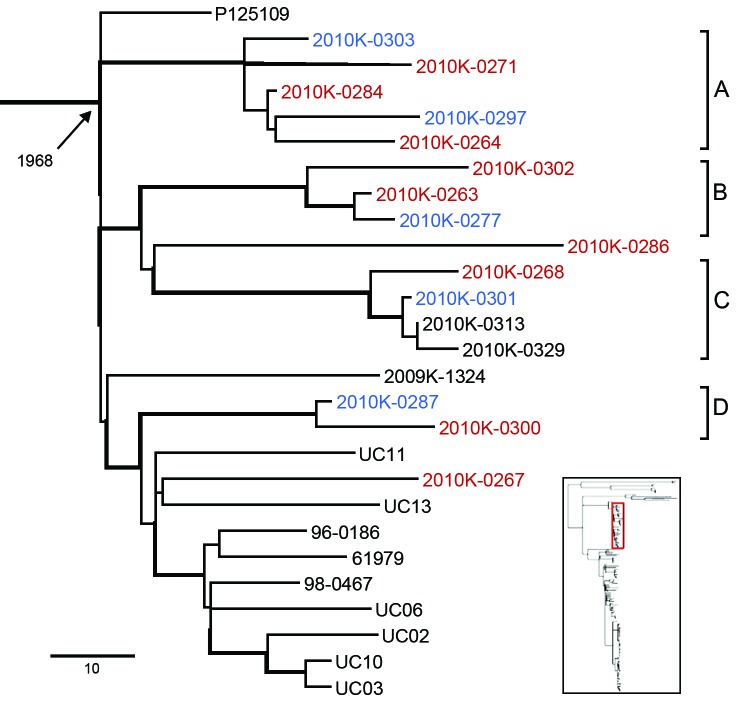
*Salmonella enterica* serotype Enteritidis genetic lineage III. Isolates from human blood and stool samples are indicated by red and blue, respectively. Four clades are highlighted and designated A–D, representing individual outbreak clades identified from clinical cases in Thailand in 2008. Branches with bootstrap value >0.9 are indicated by thickened lines. Age of the ancestral node (median most recent common ancestor) is labeled. Inset indicates the location of the highlighted lineage on the global phylogenetic tree.

Our retrospective investigation of clinical cases from Thailand and a shelled eggs outbreak in the United States demonstrated the utility of our method on the basis of the robust bioinformatics pipeline and the broad phylogenetic framework. Fourteen of the 15 isolates from clinical cases in Thailand clustered in LIII and fell into 4 different clusters with high bootstrapping support (Figure 3, clusters A–D), suggesting multiple genetic origins consistent with a previous study (*34*). Isolates from blood and fecal samples clustered with other isolates from the United States and Europe, including the reference strain P125109 from United Kingdom. Previous observation of a disproportionately high percentage of bloodstream infections of *S. enterica* Enteritidis in Thailand (*35*) may be due to host factors (e.g., underlying health conditions, concurrent infections), underreported or unreported gastrointestinal infections less detectable than invasive ones, and/or random invasive infections (e.g., high-dosage exposure). Similarly, compromised immunity from the high percentage of HIV cases in sub-Saharan Africa was proposed as a contributing factor to the perceived high invasiveness of serotype Enteritidis infections in the region (*36*). We recommend caution when interpreting extraintestinal infections of *Salmonella* as evidence for high virulence and that newly proposed hypervirulent lineages (*37*) be evaluated within a global phylogenetic context for their evolutionary identity and origin.

**Figure 4:**
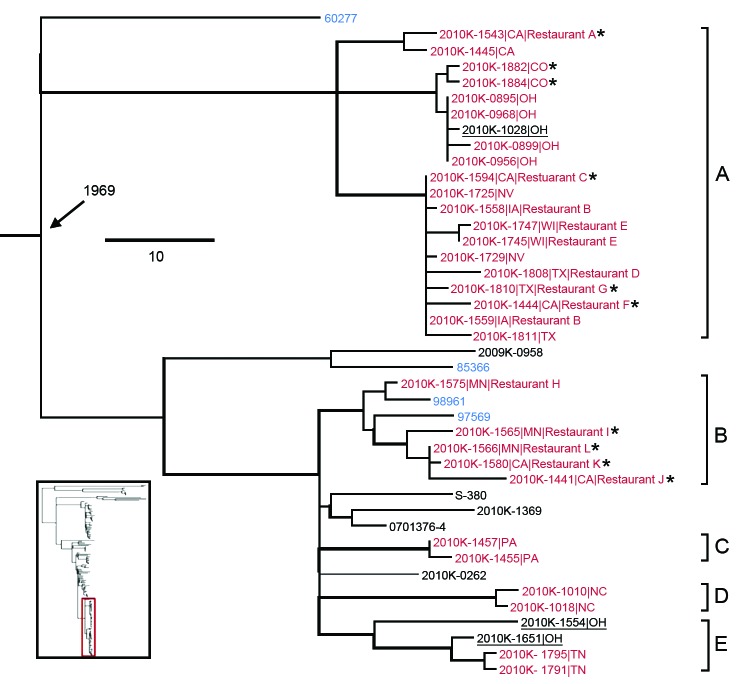
*Salmonella enterica* serotype Enteritidis clades associated with the 2010 US shelled eggs outbreak. Red indicates isolates sequenced as part of the outbreak investigation using Roche 454 technology (Indianapolis, IN, USA); blue indicates isolates associated with chicken or chicken products; asterisk (*) indicates the isolate was traced back to the implicated egg production facility. Five outbreak clades are highlighted and designated as A–E, of which A and B are definite and C, D, and E are putative. Isolates ascribed to the egg outbreak in this study are underlined. Branches with bootstrap values >0.9 are shown by thickened lines. Age of the ancestral node (median most recent common ancestor) is labeled. Scale bar indicates single-nucleotide polymorphisms. Inset indicates the location of the highlighted lineage on the global phylogenetic tree.

Concerning the shelled eggs outbreak, 2 definite (Figure 4, clusters A and B, both including isolates traced to the implicated facility) and 3 putative outbreak clades (clusters C–E, none of which had a direct epidemiologic link to the outbreak), i.e., individual clusters each containing isolates originated from a single source of contamination, were identified with phylogenetic and/or epidemiologic support on the basis of 2 criteria: 1) the clade is phylogenetically highly supported and 2) the isolation dates of all the isolates in the clade correspond to the outbreak period. Three isolates from sporadic cases in 2009 and 2010 might be attributed to the outbreak because they fell into the outbreak clades A and E (Figure 4, underlined), suggesting that the outbreak strains might have been circulating before the outbreak. Although A corresponded to a major clade defined by Allard et al. (*12*), B, C, and D clustered and thus were designated as a single clade in that study, possibly because of the absence of reference strains to separate them. Among the 4 isolates associated with poultry (Figure 4, blue highlighted), 60277 and 85366 were respectively isolated in 2002 and 2007 and therefore unlikely to be associated with each other and with the outbreak in 2010. These and other isolates unrelated to the outbreak helped delineate the individual outbreak clades, corroborating the likely polyclonal nature of the outbreak, which also was recognized by Allard et al. (*12*), and emphasizing the importance of incorporating multiple proper background references into outbreak investigations.

During outbreak investigations, putative outbreak isolates are analyzed with epidemiologically unrelated strains to determine whether they are related and thus likely to be associated with the same source. The availability and selection of background references can be critical (Technical Appendix Figure 4). To maximize the availability of suitable background reference datasets, researchers desire phylogenetic frameworks with sufficient coverage of the genetic diversity in major pathogens, which is an aim of the ongoing 100K pathogen genome project (http://100kgenome.vetmed.ucdavis.edu/index.cfm).

Phylogenetic data alone are insufficient for defining an outbreak. Determining the polyclonal nature and scope of an outbreak remains a challenge, and no definitive criteria yet exist. For example, investigations of a recent *S. enterica* serotype Montevideo outbreak associated with red and black peppers reached discrepant conclusions. A proposed domestic source isolate from the implicated food processing facility (*38*) was excluded from the outbreak clade defined in another study (*18*), presumably because of differences in the definition of the scope of the outbreak between the 2 studies. Although a polyclonal outbreak is among the explanations for this discrepancy, the possibility of other scenarios cannot be rejected by available phylogenomic and epidemiologic evidence. For instance, in a case of continuous or intermittent outbreak, a single persistent founder strain can shed divergent descendants that contaminate food and cause disease over an extended time, as reported by Orsi et al. (*39*). Such microevolution events give rise to clones that might or might not be considered as distinct genotypes or separate outbreak clades depending on levels of mutation, epidemiologic information, or even subjective interpretation. Therefore, case-by-case understanding of evolutionary dynamics and population structure of major foodborne pathogens (*40*), which might vary among different species and be affected by particular food-processing environments and outbreak vehicles, is necessary for elucidating population genetics and transmission dynamics of foodborne pathogens and lays the groundwork for the increasing application of genomic epidemiology in pathogen surveillance and outbreak investigation. Ultimately, if some strains in an outbreak have continued to evolve quickly, then the ability to cluster strains and identify outbreaks will rely even more on a suitable set of outgroups.

Technical Appendix Isolates used in this study; new genomes sequenced for this study; regions flagged as putatively involved in recombination by BratNextGen and excluded from analyses; comparison of 4 models used for phylogenetic analysis; dendrogram of top 30 most prevalent *Xba*l PFGE patterns and patterns selected for sequencing in this study; bioinformatics pipeline for single-nucleotide polymorphism detection; core genome single-nucleotide polymorphism maximum likelihood tree of various *Salmonella* serotypes; and importance of reference isolates for whole-genome sequence typing.
