# Supplementary material for: Genomic Epidemiology of Salmonella enterica Serotype Enteritidis based on Population Structure of Prevalent Lineages
Source: Emerg Infect Dis. 2014 Sep;20(9):1481–9. doi: 10.3201/eid2009.131095 (PMC4178404; doi:10.3201/eid2009.131095)
Supplement: Technical Appendix — Isolates used in this study; new genomes sequenced for this study; regions flagged as putatively involved in recombination by BratNextGen and excluded from analyses; comparison of 4 models used for phylogenetic analysis; dendrogram of top 30 most prevalent Xbal PFGE patterns and patterns selected for sequencing in this study; bioinformatics pipeline for single-nucleotide polymorphism detection; core genome single-nucleotide polymorphism maximum likelihood tree of various Salmonella serotypes; and importance of reference isolates for whole-genome sequence typing. [file 13-1095-Techapp-s1.pdf]

Article DOI: http://dx.doi.org/10.3201/eid2009.131095

Genomic Epidemiology of *Salmonella enterica* Serotype Enteritidis based on Population Structure of Prevalent Lineages

Technical Appendix

Technical Appendix Table 1. Isolates used in this study*

Isolate†	Serotype	Location	PFGE-*Xba*I‡	Year	Source	Sequencer
2009K-0477 (1)	Enteritidis	Uganda	JEGX01.0767	2009	Human	Illumina
2009K-0479 (2)	Enteritidis	Uganda	JEGX01.0767	2009	Human	Illumina
77320 (3)	Enteritidis	Western USA	JEGX01.0031	2004	Animal (deer)	Illumina
81748 (4)	Enteritidis	Western, USA	JEGX01.0048	2005	Animal (wild mammal)	Illumina
04–0307 (5)	Enteritidis	Arizona, USA	NA	2004	Almond processing plant	Illumina
J0915 (6)	Enteritidis	California, USA	NA	2001	Environment (drag swab)	Illumina
J0903 (7)	Enteritidis	California, USA	NA	2001	Environment (drag swab)	Illumina
J0828 (8)	Enteritidis	NA	NA	2001	Human	Illumina
UC12 (9)	Enteritidis	California, USA	NA	2002	Human blood	Illumina
0811210F (10)	Enteritidis	California, USA	NA	2008	Animal (sea lion)	Illumina
9810102B (11)	Enteritidis	California, USA	NA	1998	Animal (sea otter)	Illumina
502571 (12)	Enteritidis	California, USA	NA	2005	Animal (sea lion)	Illumina
CDC-STK-1280 (13)	Nitra	Slovakia	NA	1965	NA	Illumina
P125109 (14)	Enteritidis	United Kingdom	NA	1988	Human	Reference
2010K-0303 (15)	Enteritidis	Thailand	JEGX01.0158	2008	Human feces	Illumina
2010K-0271 (16)	Enteritidis	Thailand	JEGX01.0008	2008	Human blood	Illumina
2010K-0284 (17)	Enteritidis	Thailand	JEGX01.0167	2008	Human blood	Illumina
2010K-0297 (18)	Enteritidis	Thailand	JEGX01.0002	2008	Human feces	Illumina
2010K-0264 (19)	Enteritidis	Thailand	JEGX01.0019	2008	Human blood	Illumina
2010K-0302 (20)	Enteritidis	Thailand	JEGX01.0158	2008	Human blood	Illumina
2010K-0263 (21)	Enteritidis	Thailand	JEGX01.0002	2008	Human blood	Illumina
2010K-0277 (22)	Enteritidis	Thailand	JEGX01.0002	2008	Human feces	Illumina
2010K-0286 (23)	Enteritidis	Thailand	JEGX01.0019	2008	Human blood	Illumina
2010K-0268 (24)	Enteritidis	Thailand	JEGX01.0158	2008	Human blood	Illumina
2010K-0301 (25)	Enteritidis	Thailand	JEGX01.0158	2008	Human feces	Illumina
2010K-0313 (26)	Enteritidis	Mauritius	JEGX01.0004	2010	Human blood	Illumina
2010K-0329 (27)	Enteritidis	Mauritius	JEGX01.0004	2010	Human feces	Illumina
2009K-1324 (28)	Enteritidis	Kansas, USA	NA	2009	Human	Illumina
2010K-0287 (29)	Enteritidis	Thailand	JEGX01.0167	2008	Human feces	Illumina
2010K-0300 (30)	Enteritidis	Thailand	JEGX01.0019	2008	Human blood	Illumina
UC11 (31)	Enteritidis	California, USA	NA	2001	Human blood	Illumina
2010K-0267 (32)	Enteritidis	Thailand	JEGX01.0019	2008	Human blood	Illumina
UC13 (33)	Enteritidis	California, USA	NA	2009	Human blood	Illumina
96–0186 (34)	Nitra	California, USA	NA	1996	Human urine	Illumina
61979 (35)	Enteritidis	Northern plains, USA	JEGX01.0002	2002	Food ready-to-eat	Illumina
98–0467 (36)	Enteritidis	Wyoming, USA	NA	1998	Human blood	Illumina
UC06 (37)	Enteritidis	NA	NA	2004	Human blood	Illumina
UC02 (38)	Enteritidis	California, USA	NA	2004	Human feces	Illumina
UC10 (39)	Enteritidis	California, USA	NA	2004	Human	Illumina
UC03 (40)	Enteritidis	California, USA	NA	2005	Human	Illumina
2009K-1726 (41)	Enteritidis	Maryland, USA	JEGX01.0009	2009	Human	Illumina
56–3991 (42) [C8]	Enteritidis	Tennessee, USA	NA	1956	Human	Roche 454
62–1976 (43) [C8]	Enteritidis	Massachusetts, USA	NA	1962	Human	Roche 454
2010K-0860 (44)	Nitra	Maryland, USA	NA	2010	Human feces	Illumina
93–0063 (45)	Enteritidis	Puerto Rico	NA	1993	Human feces	Illumina
2010K-2029 (46)	Enteritidis	Illinois, USA	JEGX01.0002	2010	Human	Illumina
2010K-0345 (47)	Enteritidis	Mauritius	JEGX01.0004	2010	Human brain abscess	Illumina
2010K-1923 (48)	Enteritidis	Maryland, USA	JEGX01.0004	2010	Human	Illumina
2010K-0351 (49)	Enteritidis	Mauritius	JEGX01.0004	2010	Human feces	Illumina

Isolate†	Serotype	Location	PFGE-*XbaI*‡	Year	Source	Sequencer
50–3079 (50) [C7]	Enteritidis	New Jersey, USA	NA	1950	NA	Roche 454
61080 (51)	Enteritidis	Northern plains, USA	JEGX01.0004	2002	Animal (cow)	Illumina
75788 (52)	Enteritidis	Northern plains, USA	JEGX01.0003	2004	Animal (reptile)	Illumina
08–1080 (53)	Enteritidis	New Mexico, USA	JEGX01.0030	2008	Human feces	Illumina
82631 (54)	Enteritidis	Southeastern, USA	JEGX01.0111	2006	Food (chicken)	Illumina
2010K-1832 (55)	Enteritidis	Oregon, USA	JEGX01.0005	2010	Human	Illumina
0804789B (56)	Enteritidis	California, USA	NA	2008	Animal (elephant seal)	Illumina
78296 (57)	Enteritidis	Northern plains, USA	JEGX01.0030	2005	Food (chicken)	Illumina
S-277 (58)	Enteritidis	NA	JEGX01.0005	2008	Environment (scald water)	Illumina
53–0407 (59) [C6]	Enteritidis	California, USA	NA	1953	Food (turkey)	Roche 454
98–9534 (60)	Enteritidis	California, USA	NA	1998	Animal (dog)	Illumina
1102933A (61)	Enteritidis	California, USA	NA	2011	Animal (elephant seal)	Illumina
77–2659 (62) [C3]	Enteritidis	South Dakota, USA	NA	1977	Human	Roche 454
93–7741 (63)	Enteritidis	California, USA	NA	1993	Animal (horse)	Illumina
02–2966 (64)	Enteritidis	California, USA	NA	2002	Animal (rodent)	Illumina
76–2651 (65) [C4]	Enteritidis	Maryland, USA	NA	1976	Human	Roche 454
54–2220 (66)	Enteritidis	Illinois, USA	NA	1954	Human feces	Illumina
78–1757 (67) [C5]	Enteritidis	Nebraska, USA	NA	1978	Human	Roche 454
50–5646 (68) [C3]	Enteritidis	Texas, USA	NA	1950	NA	Roche 454
77–0424 (69) [C3]	Enteritidis	Arizona, USA	NA	1977	Human	Roche 454
81–2490 (70) [C3]	Enteritidis	New Jersey, USA	NA	1981	Human	Roche 454
84–1226 (71)	Enteritidis	New York, USA	NA	1984	Human urine	Illumina
35986 (72)	Enteritidis	NA	JEGX01.0021	1998	Food (chicken)	Illumina
81–2625 (73) [C3]	Enteritidis	New Mexico, USA	NA	1981	Human	Roche 454
39997 (74)	Enteritidis	Southeastern, USA	JEGX01.0037	1999	Food (chicken)	Illumina
76–3618 (75) [C3]	Enteritidis	Arizona, USA	NA	1976	Human	Roche 454
76–0331 (76)	Enteritidis	Saudi Arabia	NA	1976	Human feces	Illumina
69–4941 (77) [C3]	Enteritidis	New Hampshire, USA	NA	1969	Human	Roche 454
77–1427 (78) [C3]	Enteritidis	Rhode Island, USA	NA	1977	Human	Roche 454
75–2732 (79)	Enteritidis	New Hampshire, USA	NA	1975	Human feces	Illumina
93–2836A (80)	Enteritidis	California, USA	NA	1993	Animal (elephant seal)	Illumina
93–6175B (81)	Enteritidis	California, USA	NA	1993	Animal (sea lion)	Illumina
93–7922A (82)	Enteritidis	California, USA	NA	1993	Animal (elephant seal)	Illumina
55795(83)	Enteritidis	Southeastern, USA	JEGX01.0005	2001	Food (chicken)	Illumina
60277 (84)	Enteritidis	Northeastern USA	JEGX01.0004	2002	Food (egg yolks)	Illumina
2010K-1543 (85) [C1]	Enteritidis	California, USA	JEGX01.0004	2010	Human	Roche 454
2010K-1445 (86) [C1]	Enteritidis	California, USA	JEGX01.0004	2010	Human	Roche 454
2010K-1882 (87) [C1]	Enteritidis	Colorado, USA	JEGX01.0005	2010	Human	Roche 454
2010K-1884 (88) [C1]	Enteritidis	Colorado, USA	JEGX01.0004	2010	Food ready-to-eat	Roche 454
2010K-0895 (89) [C1]	Enteritidis	Ohio, USA	JEGX01.0004	2010	Human	Roche 454
2010K-0968 (90) [C1]	Enteritidis	Ohio, USA	JEGX01.0004	2010	Human	Roche 454
2010K-1028 (91)	Enteritidis	Ohio, USA	JEGX01.0004	2010	Human	Illumina
2010K-0899 (92) [C1]	Enteritidis	Ohio, USA	JEGX01.0004	2010	Food ready-to-eat	Roche 454
2010K-0956 (93) [C1]	Enteritidis	Ohio, USA	JEGX01.0004	2010	Human	Roche 454
2010K-1594 (94) [C1]	Enteritidis	California, USA	JEGX01.0004	2010	Human	Roche 454
2010K-1725 (95) [C1]	Enteritidis	Nevada, USA	JEGX01.0004	2010	Human	Roche 454
2010K-1558 (96) [C1]	Enteritidis	Iowa, USA	JEGX01.0004	2010	Human	Roche 454
2010K-1747 (97) [C1]	Enteritidis	Wisconsin, USA	JEGX01.0004	2010	Human	Roche 454
2010K-1745 (98) [C1]	Enteritidis	Wisconsin, USA	JEGX01.0004	2010	Human	Roche 454
2010K-1729 (99) [C1]	Enteritidis	Nevada, USA	JEGX01.0004	2010	Human	Roche 454
2010K-1808 (100) [C1]	Enteritidis	Texas, USA	JEGX01.0004	2010	Human	Roche 454
2010K-1810 (101) [C1]	Enteritidis	Texas, USA	JEGX01.0004	2010	Human	Roche 454
2010K-1444 (102) [C1]	Enteritidis	California, USA	JEGX01.0004	2010	Human	Roche 454
2010K-1559 (103) [C1]	Enteritidis	Iowa, USA	JEGX01.0004	2010	Human	Roche 454
2010K-1811 (104) [C1]	Enteritidis	Texas, USA	JEGX01.0004	2010	Human	Roche 454
2009K-0958 (105)	Enteritidis	Massachusetts, USA	JEGX01.0021	2009	Human	Illumina
85366 (106)	Enteritidis	Western USA	JEGX01.0021	2007	Food (ground chicken)	Illumina
2010K-1575 (107) [C2]	Enteritidis	Minnesota, USA	JEGX01.0004	2010	Human	Roche 454
98961 (108)	Enteritidis	Southeastern USA	JEGX01.0004	2010	Food (chicken	Illumina
97569 (109)	Enteritidis	Northern plains, USA	JEGX01.0004	2010	Food (ground chicken)	Illumina
2010K-1565 (110) [C2]	Enteritidis	Minnesota, USA	JEGX01.0004	2010	Human	Roche 454
2010K-1566 (111) [C2]	Enteritidis	Minnesota, USA	JEGX01.0004	2010	Human	Roche 454
2010K-1580 (112) [C2]	Enteritidis	Minnesota, USA	JEGX01.0004	2010	Human	Roche 454
2010K-1441 (113) [C2]	Enteritidis	California, USA	JEGX01.0004	2010	Human	Roche 454
S-380 (114)	Enteritidis	NA	JEGX01.0003	2008	Environment (foam)	Illumina
2010K-1369 (115)	Enteritidis	Iowa, USA	JEGX01.0034	2010	Human feces	Illumina

Isolate†	Serotype	Location	PFGE-*Xba*I‡	Year	Source	Sequencer
0701376–4 (116)	Enteritidis	California, USA	NA	2007	Animal (sea otter)	Illumina
2010K-1457 (117) [C2]	Enteritidis	Pennsylvania, USA	JEGX01.0004	2010	Human	Roche 454
2010K-1455 (118) [C2]	Enteritidis	Pennsylvania, USA	JEGX01.0004	2010	Human	Roche 454
2010K-0262 (119)	Enteritidis	Thailand	JEGX01.0019	2008	Human feces	Illumina
2010K-1010 (120) [C2]	Enteritidis	North Carolina, USA	JEGX01.0108	2010	Food ready-to-eat	Roche 454
2010K-1018 (121) [C2]	Enteritidis	North Carolina, USA	JEGX01.0004	2010	Human	Roche 454
2010K-1554 (122)	Enteritidis	Iowa, USA	JEGX01.0004	2010	Human	Illumina
2009K-1651 (123)	Enteritidis	California, USA	JEGX01.0034	2009	Human	Illumina
2010K-1795 (124) [C2]	Enteritidis	Tennessee, USA	JEGX01.0004	2010	Human	Roche 454
2010K-1791 (125) [C2]	Enteritidis	Tennessee, USA	JEGX01.0004	2010	Human	Roche 454
77–0915§	Enteritidis	New Zealand	NA	1977	NA	Illumina
07–0056§	Enteritidis	Minnesota, USA	NA	2006	Human blood	Illumina
SARB17§	Enteritidis	Brazil	NA	NA	NA	454/Sanger¶
SARB19§	Enteritidis	Switzerland	NA	NA	NA	Illumina¶

*PFGE, pulsed-field gel electrophoresis; NA, not available,

†Isolates are numbered (in parenthesis) from the top down as they appear on the 125-isolate tree in Figure 1. For each 454 sequenced isolate also analyzed in (*12*), its cluster affiliation is noted in brackets as defined in (*12*).‡PulseNet PFGE pattern names might be subject to change.

§Genetically divergent isolates that are excluded from phylogenetic analysi.

¶Desai PT, Porwollik S, Long F, Cheng P, Wollam A, Bhonagiri-Palsikar V, et al. Evolutionary genomics of *Salmonella enterica* subspecies. mBio. 2013;4:e00198–13.

Technical Appendix Table 2. New genomes sequenced for this study

Isolate	GenBank accession no.	Sequencing coverage
UC07	PRJNA168935	64.5×
07–0056	PRJNA168933	61.2×
60277	PRJNA168942	64.8×
97569	PRJNA168772	63.6×
85366	PRJNA168767	55.6×
61979	PRJNA168769	64.3×
98961	PRJNA168768	97.6×
78296	PRJNA168766	57.4×
82631	PRJNA168792	66.7×
81748	PRJNA168793	99.0×
77320	PRJNA168794	90.1×
61080	PRJNA168770	75.2×
39997	PRJNA168791	64.0×
55795	PRJNA168764	77.0×
34986	PRJNA168771	88.8×
2010K-0313	PRJNA168735	56.0×
2010K-0329	PRJNA168736	56.5×
2010K-0345	PRJNA168753	64.8×
2010K-0351	PRJNA168754	50.7×
77–0915	PRJNA168760	60.7×
76–0331	PRJNA168759	65.2×
2010K-0284	PRJNA168728	79.8×
2010K-0287	PRJNA168730	60.2×
2010K-0297	PRJNA168731	56.2×
2010K-0300	PRJNA168732	55.6×
2010K-0302	PRJNA168733	95.5×
2010K-0303	PRJNA168734	60.5×
2010K-0268	PRJNA168750	61.0×
2010K-0301	PRJNA168752	98.3×
2010K-0271	PRJNA168727	52.6×
2010K-0286	PRJNA168729	98.2×
2010K-0264	PRJNA168749	59.2×
2010K-0262	PRJNA168725	60.5×
2010K-0267	PRJNA168726	66.2×
2010K-0263	PRJNA168748	55.7×
2010K-0277	PRJNA168751	51.9×
2009K-0477	PRJNA168745	61.9×
2009K-0479	PRJNA168746	57.9×
04–0307	PRJNA168744	57.6×
2009K-1651	PRJNA168723	57.6×
J0903	PRJNA168762	69.2×
J0915	PRJNA168795	56.1×

Isolate	GenBank accession no.	Sequencing coverage
93–6175B	PRJNA168773	95.6×
93–2836A	PRJNA168774	98.7×
93–7741	PRJNA168775	93.9×
93–7922A	PRJNA168776	92.3×
98–9534	PRJNA168777	99.4×
02–2966	PRJNA168778	98.7×
502571	PRJNA168779	98.6×
0701376–4	PRJNA168780	99.0×
0804789B	PRJNA168781	98.6×
0811210F	PRJNA168782	99.1×
1102933A	PRJNA168783	99.4×
9810102B	PRJNA168784	99.4×
54–2220	PRJNA168739	57.2×
2010K-1554	PRJNA168757	99.4×
2009K-1324	PRJNA168747	65.0×
2009K-1726	PRJNA168724	67.6×
2010K-1923	PRJNA168737	64.6×
75–2732	PRJNA168740	57.4×
08–1080	PRJNA168721	96.8×
84–1226	PRJNA168741	56.4×
2010K-1028	PRJNA168755	98.6×
2010K-1832	PRJNA168758	63.3×
93–0063	PRJNA168742	95.5×
UC02	PRJNA168785	49.8×
UC03	PRJNA168786	55.7×
UC10	PRJNA168787	59.5×
UC11	PRJNA168788	62.4×
UC12	PRJNA168789	56.8×
UC13	PRJNA168790	60.8×
2009K-0958	PRJNA168722	56.7×
2010K-1369	PRJNA168756	55.4×
98–0467	PRJNA168743	49.5×
S-380	PRJNA168763	74.1×
S-277	PRJNA168765	69.7×
2010K-2029	PRJNA168738	74.7×
J0828	PRJNA168761	66.2×
CDC-STK −1280	PRJNA168845	47.6×
96–0186	PRJNA168844	54.2×
2010K-0860	PRJNA168843	56.3×
UC06	PRJNA168940	64.2×

Appendix Table 3. Regions flagged as putatively involved in recombination by BratNextGen (Helsinki, Finland) and excluded from analyses

Region	Start	Stop	Locus tags in the reference genome
1	1	31813	SEN0001-SEN0027
2	66159	69974	SEN0057-SEN0061
3	468453	471763	SEN0418-SEN0420
4	570412	590805	SEN0509-SEN0530
5	774386	774422	SEN0697
6	784432	812238	SEN0706-SEN0732
7	924608	926567	SEN0841
8	1000063	1044312	SEN0899-SEN0940
9	1602387	1725731	SEN1501-SEN1621
10	1986102	2093597	SEN1881-SEN2005
11	3402494	3402716	SEN3185
12	4070069	4070244	SEN_r016
13	4232858	4403391	SEN3930-SEN4072
14	4576106	4578296	SEN4254

*P125109; NCBI reference sequence: NC_011294.1.

Technical Appendix Table 4. Comparison of 4 models used for phylogenetic analysis*

	Marginal likelihood	Model 1	Model 2	Model 3	Model 4
Model 1	–34183.35	**–**	**79.14**	**–172.99**	**–290.17**
Model 2	–34262.49	**–79.14**	**–**	**–252.13**	**–369.31**
Model 3	–34010.36	**172.99**	**252.13**	**–**	**–117.18**
Model 4	–33893.18	**290.17**	**369.31**	**117.18**	**–**

*Marginal likelihoods and Bayes factors (in bold) are shown. Model 1: strict clock and constant effective populations size; Model 2: strict clock and Gaussian Markov random field (GMRF) skyride; Model 3: relaxed clock and GMRF skride; Model 4: relaxed clock and constant effective population size. For Bayesian phylogenetic analysis, isolation year of each isolate was used as the tip date to calibrate estimated phylogenetic trees. Three different model combinations were tested: 1) strict molecular clock (applying a constant substitution rate across the entire tree) with constant effective populations size; 2) strict molecular clock with constant effective population size; and 3) lognormal relaxed clock (allowing for different substitution rates on different lineages) with GMRF skyride model; and 4) lognormal relaxed clock with constant effective population size. Every combination was run in 3 independent replicates for 100 million generations each, and model parameters and trees were logged every 10,000 generations. The results of the first 15 million generations (roughly the burn-in for most runs) were discarded. If convergence and proper mixing (as confirmed by Tracer 1.5, http://tree.bio.ed.ac.uk/software/tracer/) was achieved for each individual run, the results of the replicate runs were combined for further analysis. For the parameter-rich GMRF model, 5 independent runs of 100 million generations were necessary to obtain proper convergence and mixing. In this case the replicate runs were combined for further analysis. Bayes factors (BF) for each model combination were calculated based on the marginal likelihood values calculated using the path sampling method.

References

1. Kislyuk AO, Katz LS, Agrawal S, Hagen MS, Conley AB, Jayaraman P, et al. A computational genomics pipeline for prokaryotic sequencing projects. Bioinformatics. 2010;26:1819–26. PubMed http://dx.doi.org/10.1093/bioinformatics/btq284

2. Langmead B, Salzberg SL. Fast gapped-read alignment with Bowtie 2. Nat Methods. 2012;9:357–9. PubMed http://dx.doi.org/10.1038/nmeth.1923

3. Li H, Handsaker B, Wysoker A, Fennell T, Ruan J, Homer N, et al. The Sequence Alignment/Map format and SAMtools. Bioinformatics. 2009;25:2078–9. PubMed http://dx.doi.org/10.1093/bioinformatics/btp352

4. Cock PJ, Antao T, Chang JT, Chapman BA, Cox CJ, Dalke A, et al. Biopython: freely available Python tools for computational molecular biology and bioinformatics. Bioinformatics. 2009;25:1422–3. PubMed http://dx.doi.org/10.1093/bioinformatics/btp163

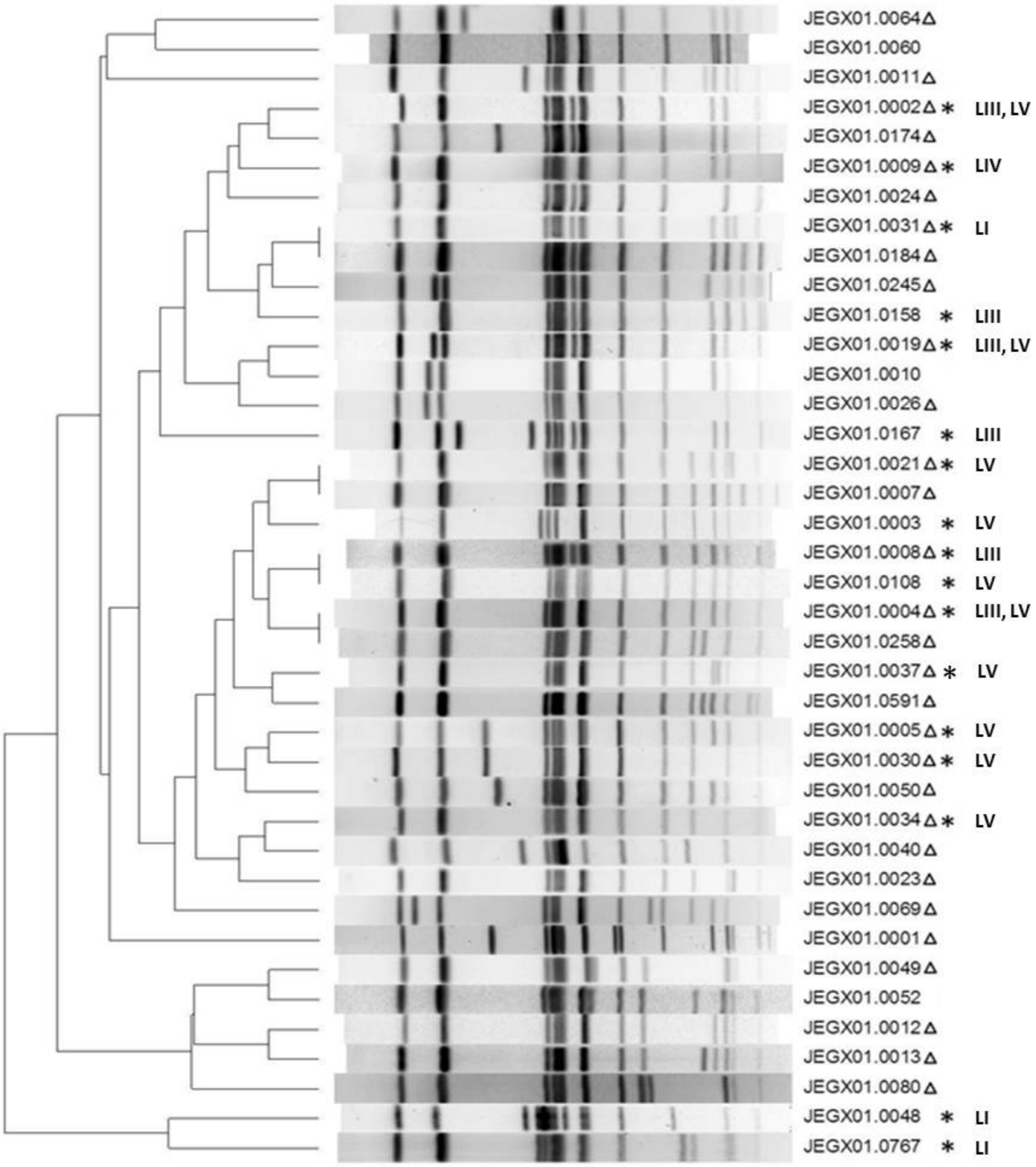

Appendix Figure 1. Dendrogram of top 30 most prevalent *Xba*I pulsed-field gel electrophoresis (PFGE) patterns and patterns selected for sequencing in this study. Δ indicates a top 30 most prevalent pattern. * indicates a sequenced pattern. Corresponding lineage(s) of each PFGE pattern is labeled.

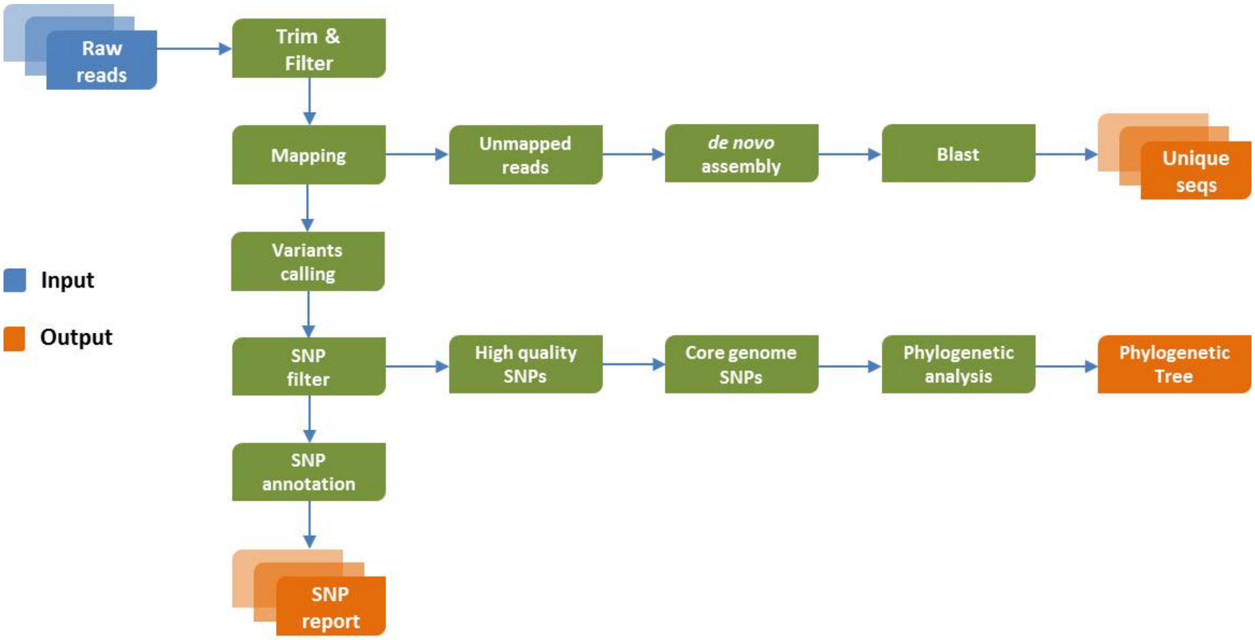

Appendix Figure 2. Bioinformatics pipeline for single-nucleotide polymorphism (SNP) detection. In this study, each raw read in fastq format was trimmed from both ends until whichever of the 2 criteria was met: 1) the end (i.e., the trimmed part) reached an average Phred quality score of 35 or 2) a maximum of 20 bp at each end was removed (*1*). After trimming, any read below an average Phred score of 30 or a minimum length of 62 bp was removed. Trimmed and filtered reads were then mapped to *Salmonella enterica* serotype Enteritidis reference genome (P125109**;** NCBI reference sequence: NC_011294.1) with Bowtie 2 using default settings (*2*). For genomes sequenced by Illumina (n = 81) and 454 (n = 44), the average depth of mapping was 60× and 14×, respectively. Variants calls (SNPs, insertions and deletions) and consensus sequences were generated from BAM files by using SAMtools Mpileup (*3*), after BAM file sorting and removal of potential PCR duplicates. For each genome analyzed, a list of high-quality SNPs was derived by subjecting initial SNP calls to a set of quality filters including a minimum Phred base score of 60, a minimum read mapping score of 20, a mapping depth ranging from 5 to 100 reads per locus, a maximum alternate allele percentage of 25% and SNPs were accepted only when confirmed by reads mapped to both the forward and reverse strands. An optional step of SNP annotation was performed to include functional information such as amino acid change if a nonsynonymous point mutation occurred in a coding region. The pipeline was written in Python by using Biopython modules (*4*). All scripts are available upon request. The program snp-sites was then used to code missing data and SNP sites from ambiguous sites within the consensus sequences and create a multifasta alignment containing variable sites (https://github.com/andrewjpage/snp_sites).

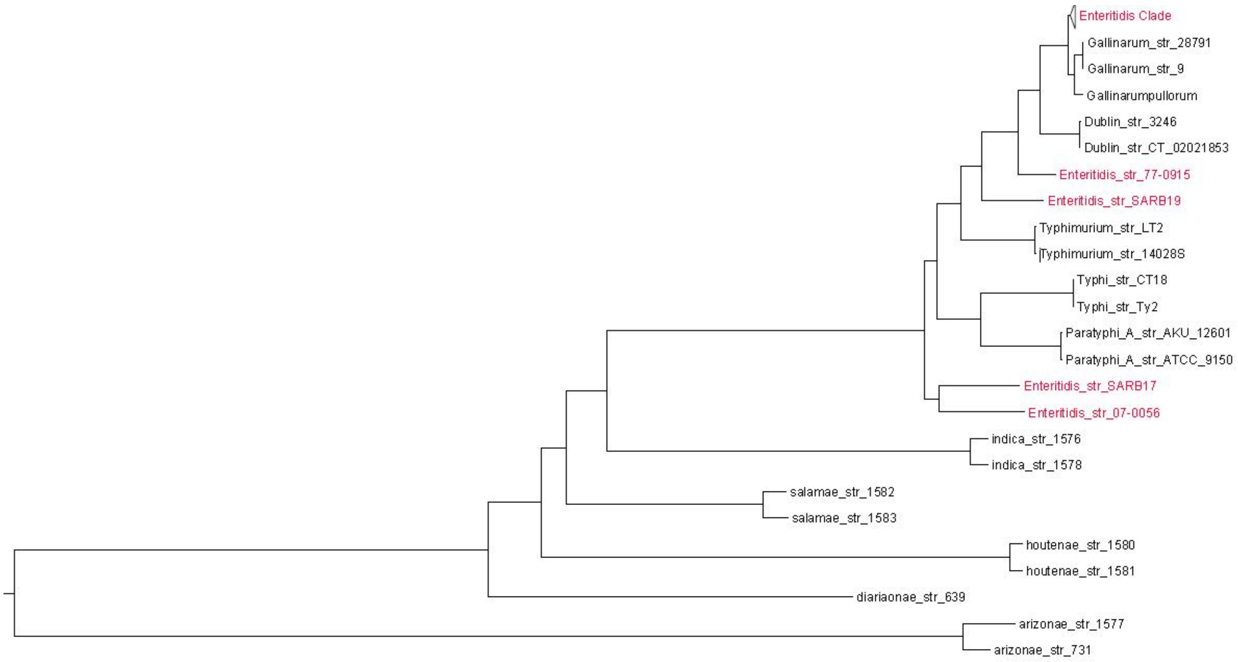

Appendix Figure 3. Core genome single-nucleotide polymorphism maximum likelihood tree of various *Salmonella* serotypes. The main *Salmonella enterica* serotype Enteritidis lineage and the 4 divergent serotype Enteritidis isolates are highlighted in red.

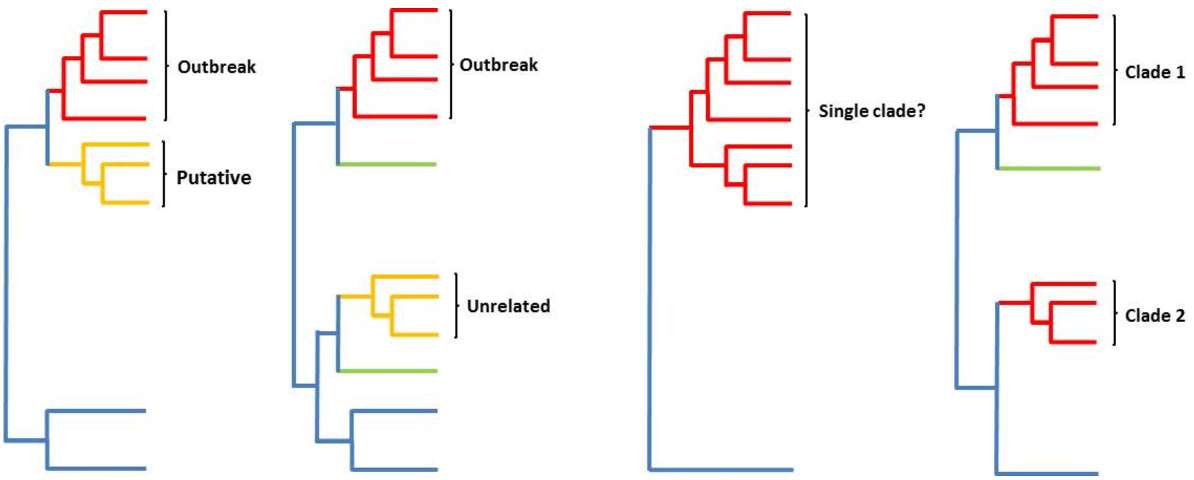

Appendix Figure 4. Importance of reference isolates for whole-genome sequence typing (WGST) outbreak investigation. The outbreak clade is shown in red. Putative and epidemiologically unrelated clades are shown in yellow. Green nodes represent reference isolates. First, the use of proper reference may help exclude isolates that are unrelated to the outbreak under investigation. Without the reference strains highlighted in green in Appendix Figure 4A, the putative outbreak clade highlighted in yellow would cluster with the outbreak clade and appear to be more genetically homogeneous that it actually is. However, with the proper reference strains in place, the yellow clade is most likely a distinct lineage. Second, co-analyzing with a proper set of reference strains can help to separate outbreak isolates into individual clades and uncover multiple sources of contamination in the case of a polyclonal outbreak where multiple strains are simultaneously involved as shown in Appendix Figure 4B. Although phylogeny may help delineate outbreaks, it should not be used as the sole basis for ascribing/excluding isolates to/from an outbreak.
